# P-1026. Predictors of Consolidations, Such as Non-Round Image Pattern, in CT Findings in Patients with Invasive Mold Pneumonia

**DOI:** 10.1093/ofid/ofae631.1216

**Published:** 2025-01-29

**Authors:** Hyeon Mu Jang, Euijin Chang, Seongman Bae, Jiwon Jung, Min Jae Kim, Yong Pil Chong, Sang-Oh Lee, Sang-Ho Choi, Yang Soo Kim, Sung-Han Kim

**Affiliations:** University of Ulsan College of Medicine (Asan Medical Center), Seoul, Republic of Korea, Seoul, Seoul-t'ukpyolsi, Republic of Korea; Asan medical center/Department of Infectious disease, Seoul, Seoul-t'ukpyolsi, Republic of Korea; Asan medical center/Department of Infectious disease, Seoul, Seoul-t'ukpyolsi, Republic of Korea; Asan Medical Center, Seoul, Seoul-t'ukpyolsi, Republic of Korea; Asan Medical Center, Seoul, Seoul-t'ukpyolsi, Republic of Korea; Asan Medical Center, Seoul, Seoul-t'ukpyolsi, Republic of Korea; Asan Medical Center, Seoul, Seoul-t'ukpyolsi, Republic of Korea; Asan Medical Center, Seoul, Seoul-t'ukpyolsi, Republic of Korea; Asan Medical Center, Seoul, Seoul-t'ukpyolsi, Republic of Korea; Asan medical center, Seoul, Seoul-t'ukpyolsi, Republic of Korea

## Abstract

**Background:**

CT is important to diagnose pulmonary invasive mold infection (PIMI). According to 2008 European Organization for Research and Treatment of Cancer (EORTC) criteria, dense, well-circumscribed lesion(s) was listed, but the recent 2020 EORTC criteria further categorized into wedge-shaped and segmental or lobar consolidation in addition to the dense, well-circumscribed lesion(s). However, there are limited data on the different clinical characteristics between these two CT findings. We thus evaluated the frequency of the newly added criterion (consolidation) depending on the underlying diseases.

**Figure:**
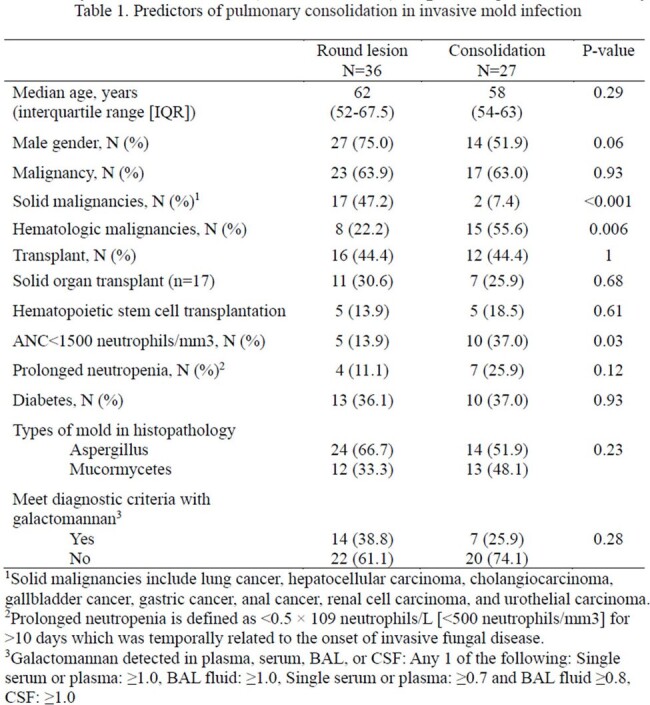

**Methods:**

We reviewed all adult patients, at a 2700-bed tertiary hospital, Seoul, South Korea, between June 2007 and December 2023, who were diagnosed with proven PIMI, based on 2020 EORTC criteria. CT findings were classified as “round lesion” if those had well-circumscribed lesions such as nodule, mass or cavity and “consolidation” if those had wedge-shaped and segmental or lobar consolidation.

**Results:**

A total of 63 patients with proven PIMIs: 38 (60.3%) aspergillosis and 25 (39.7%) mucormycosis. Of these 63 patients, 19 (30.2%) had a solid malignancy (SM), 18 (28.6%) underwent solid organ transplant and 23 (36.5%) had a hematologic malignancy (HM) including 10 hematopoietic stem cell transplant recipients. Of these, 27 (43%) patients had consolidations in CT findings, and the remaining had “round lesion”. Consolidation was significantly more frequent in patients with neutropenia (< 1500 neutrophils/mm^3^) (33.3% [5/15] vs 66.7% [10/15]; p 0.03). It was also significantly more common in HM (34.8% [8/23] vs 65.2% [15/23]; p 0.0007) but less in SM (89.5% [17/19] vs 10.5% [2/19]; p 0.006).

**Conclusion:**

More than 40% of proven PIMI showed lung consolidation, which is more frequent in neutropenia and HM but less common in SM.

**Disclosures:**

**All Authors**: No reported disclosures

